# Pleural desmoid tumor mimicking a myofibroblastic neoplasm: Imaging-pathologic correlation

**DOI:** 10.1016/j.radcr.2026.07.114

**Published:** 2026-08-26

**Authors:** Melissa Villegas-Albo, Aldo Mijail Pacheco-Carrillo, Diego Salinas-Rodriguez, Alfredo Elizondo-Montemayor, Erwin Jesús Narváez-García, Luis Alberto Ortega-Zapata, Matías Salinas-Chapa, Guillermo Elizondo-Riojas, José Daniel Zazueta-León, Hayde Sarahi Ramos-Marrero, Natalia Vilches-Cisneros

**Affiliations:** a Department of Radiology, Centro Universitario de Imagen Diagnóstica, Hospital Universitario “Dr. José Eleuterio González”, Universidad Autónoma de Nuevo León, Monterrey, México; b Department of Urology, EMCS Programa Multicéntrico de Especialidades Médicas TecSalud, Hospital Metropolitano “Dr. Bernardo Sepúlveda”, Monterrey, Mexico; c Department of Pathology and Cytopathology, Hospital Universitario “Dr. José Eleuterio González”, Universidad Autónoma de Nuevo León, Monterrey, México

**Keywords:** Desmoid tumor, Fibromatosis, Aggressive, Pleural neoplasms, Thoracic wall, Immunohistochemistry

## Abstract

Desmoid tumors are rare, locally aggressive soft-tissue neoplasms that may present a diagnostic challenge due to overlapping imaging and histopathologic features with other myofibroblastic lesions. We report the case of a 41-year-old woman presenting with a 2-year history of right-sided chest pain and progressive numbness of the right upper extremity. Contrast-enhanced computed tomography (CT) demonstrated a large pleural-based mass with chest wall extension. Initial biopsy suggested an inflammatory myofibroblastic tumor; however, further characterization with magnetic resonance imaging (MRI) and subsequent surgical resection were performed. Definitive diagnosis was established by histopathologic and immunohistochemical analysis, confirming desmoid-type fibromatosis. Despite partial symptom improvement after surgery, follow-up imaging at 30 months demonstrated persistent soft-tissue involvement at the thoracic inlet with tumor progression, correlating with ongoing neurologic symptoms. This case highlights the importance of radiologic–pathologic correlation for accurate diagnosis and emphasizes the locally aggressive behavior and recurrence potential of pleural desmoid tumors, particularly when critical structures are involved.

## Introduction

Desmoid tumors, also known as deep or aggressive fibromatosis, are rare mesenchymal neoplasms arising from fibroblastic or myofibroblastic elements [1,2]. They account for approximately 3.5% of all fibrous tumors and less than 0.03% of all neoplasms, with an estimated annual incidence of 2-4 cases per million individuals in the general population [1,3]. These tumors show a slight female predominance and most commonly occur during the third and fourth decades of life [1].

According to the World Health Organization classification of soft tissue tumors, desmoid-type fibromatosis is categorized as an intermediate (locally aggressive) tumor with fibroblastic/myofibroblastic differentiation [2]. Despite their benign histologic appearance and lack of metastatic potential, these lesions are characterized by slow but progressive growth, local invasion, and a high rate of recurrence following surgical resection [3].

The pathogenesis of desmoid tumors remains incompletely understood and is likely multifactorial, with proposed contributing factors including prior trauma, surgical interventions, hormonal influences such as estrogen, and genetic predisposition [3]. Based on their anatomical location, they are broadly classified as intra-abdominal, abdominal wall, or extra-abdominal tumors, although they may arise in virtually any part of the body [1].

Clinically, desmoid tumors typically present as firm, infiltrative masses that may remain asymptomatic for prolonged periods, particularly in deep locations. Imaging findings are often nonspecific, frequently demonstrating ill-defined lesions with a fibrous component on computed tomography (CT). However, definitive diagnosis relies on histopathologic evaluation with immunohistochemical correlation, which remains the gold standard [3].

Intrapleural desmoid tumors, in particular, tend to remain clinically silent until they reach a considerable size, at which point symptoms such as chest pain, dyspnea, or neurologic manifestations may arise due to invasion of the chest wall or compression of adjacent structures [1]. In this report, we present a case of pleural desmoid tumor with extension into the right superior thoracic inlet, highlighting its imaging features, diagnostic challenges, and clinical implications.

## Case report

A 41-year-old woman presented with a 2-year history of right-sided chest pain radiating to the ipsilateral arm, associated with progressive numbness of the right upper extremity. Symptoms were persistent, with a reported pain intensity of 6/10. Due to ongoing symptoms, a chest radiograph was obtained, revealing a right-sided thoracic mass.

**Fig. 1:**
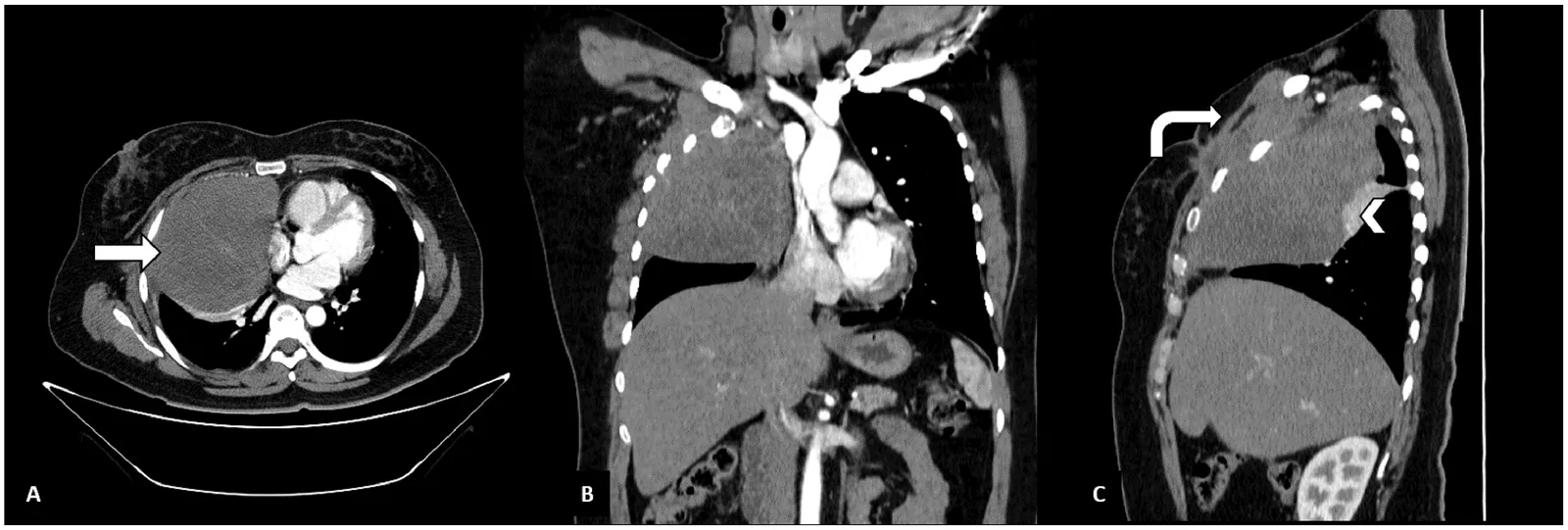
Arterial-phase contrast-enhanced chest CT showing a large right pleural-based mass. (A) Axial image demonstrates a well-defined heterogeneous mass measuring approximately 14 × 9 × 15 cm (arrow), with partial right upper and middle lobe atelectasis and leftward cardiac displacement. (B) Coronal reconstruction shows its craniocaudal extent and mass effect. (C) Sagittal reconstruction demonstrates extension into the anterior chest wall and infraclavicular soft tissues (curved arrow) and compressive atelectasis (arrowhead).

**Fig. 2:**
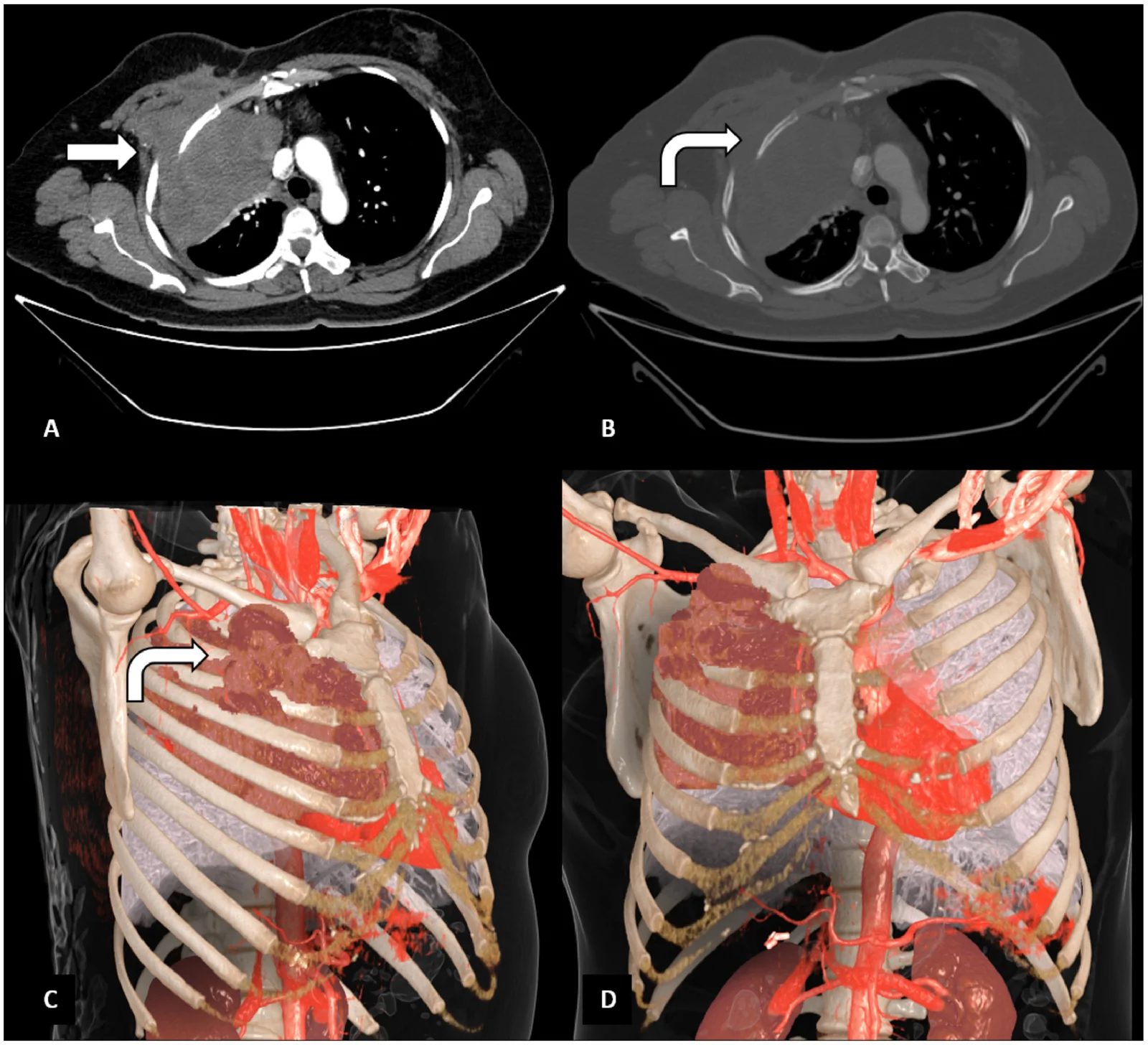
Arterial-phase contrast-enhanced chest CT showing chest wall involvement. (A) Axial soft-tissue window demonstrates tumor extension most prominent between the second and third costal arches (arrow). (B) Bone window shows preserved cortical bone without osseous destruction. (C and D) Oblique and coronal cinematic-rendering images demonstrate extrathoracic extension along the anterior chest wall (curved arrow).

Subsequent contrast-enhanced chest computed tomography (CT) demonstrated a large right pleural-based mass with heterogeneous soft-tissue attenuation, causing compression of adjacent lung parenchyma and mediastinal displacement (Fig. 1). Further evaluation showed extension into the anterior chest wall without evidence of osseous destruction, better characterized on multiplanar reconstructions and cinematic rendering images (Fig. 2).

**Fig. 3:**
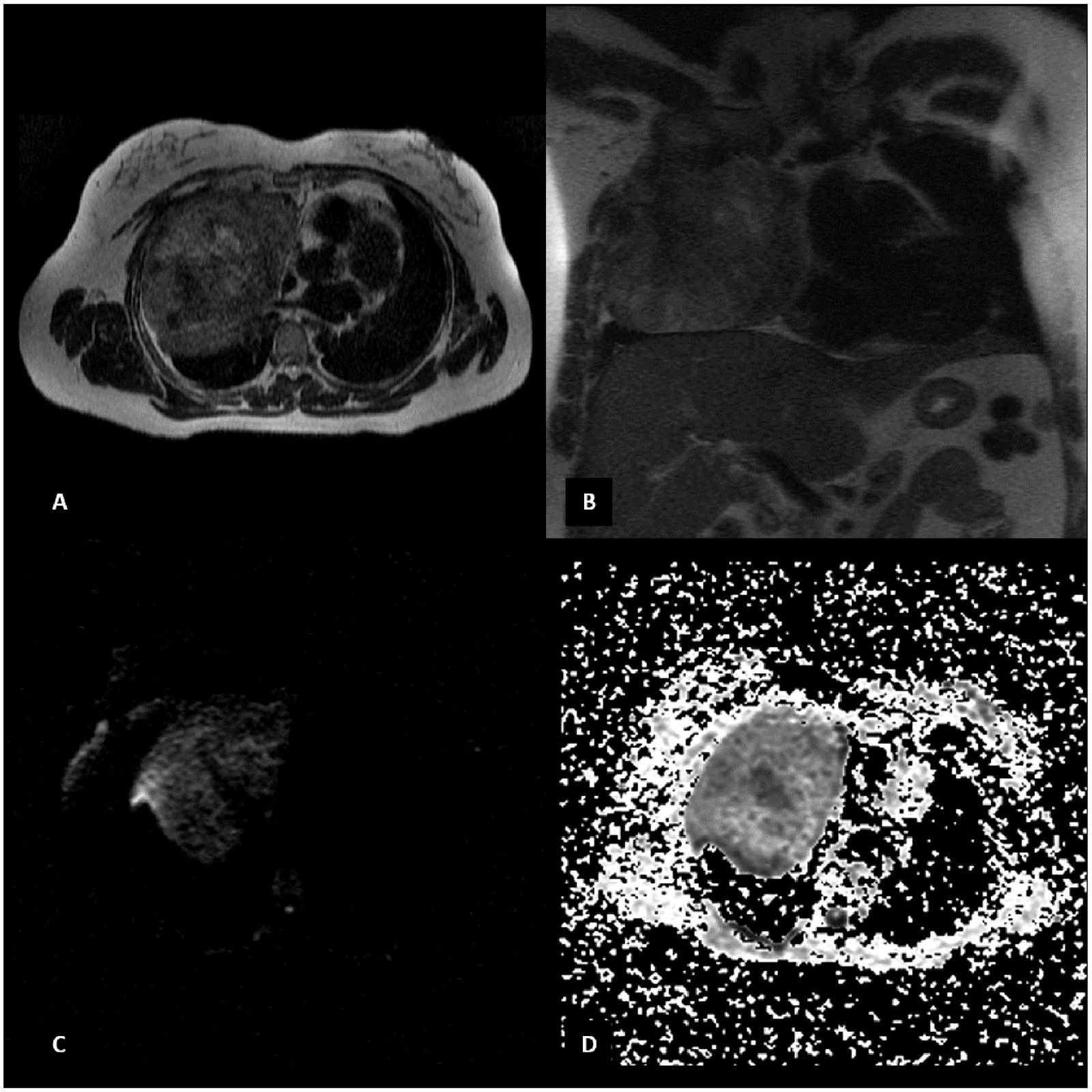
Preoperative MRI obtained 4 days after admission. (A and B) T2-weighted single-shot fast spin-echo (SSFSE) images show a pleural-based mass with heterogeneous, predominantly intermediate signal intensity. (C) Diffuse-weighted imaging (DWI) at b = 800 s/mm² demonstrates heterogeneous restricted diffusion. (D) The corresponding apparent diffusion coefficient (ADC) map confirms diffusion restriction.

A percutaneous biopsy was then performed, and histopathologic analysis initially suggested an inflammatory myofibroblastic tumor based on smooth muscle actin positivity and myofibroblastic proliferation, while β-catenin expression was not evaluated or reported. Based on these findings, surgical management was considered, and preoperative magnetic resonance imaging (MRI) was requested for improved soft-tissue characterization. MRI performed 4 days after admission demonstrated a pleural-based lesion with heterogeneous, predominantly intermediate signal intensity on T2-weighted sequences and areas of restricted diffusion on diffusion-weighted imaging (Fig. 3).

**Fig. 4:**
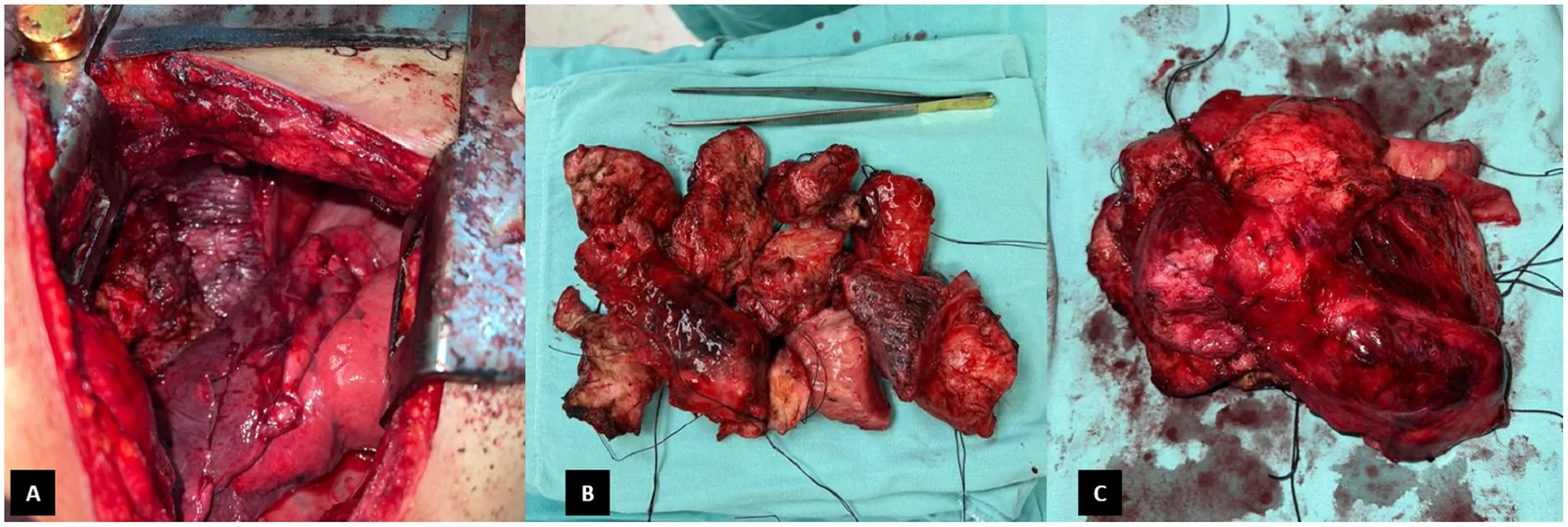
Intraoperative and gross surgical findings. (A) Intercostal surgical approach showing the tumor involving the pleural space and adjacent structures. (B and C) Gross specimens demonstrate a firm, irregular mass removed in multiple fragments because of its dense fibrous consistency.

Surgical resection was subsequently performed through a right thoracotomy, achieving partial tumor removal without complications. Complete excision was not considered feasible because the lesion demonstrated extensive infiltration of the pleural space, adjacent chest wall structures, and surrounding tissues, and complete resection would have required substantial functional sacrifice. The tumor r was resected in multiple fragments due to its dense fibrous consistency (Fig. 4).

**Fig. 5:**
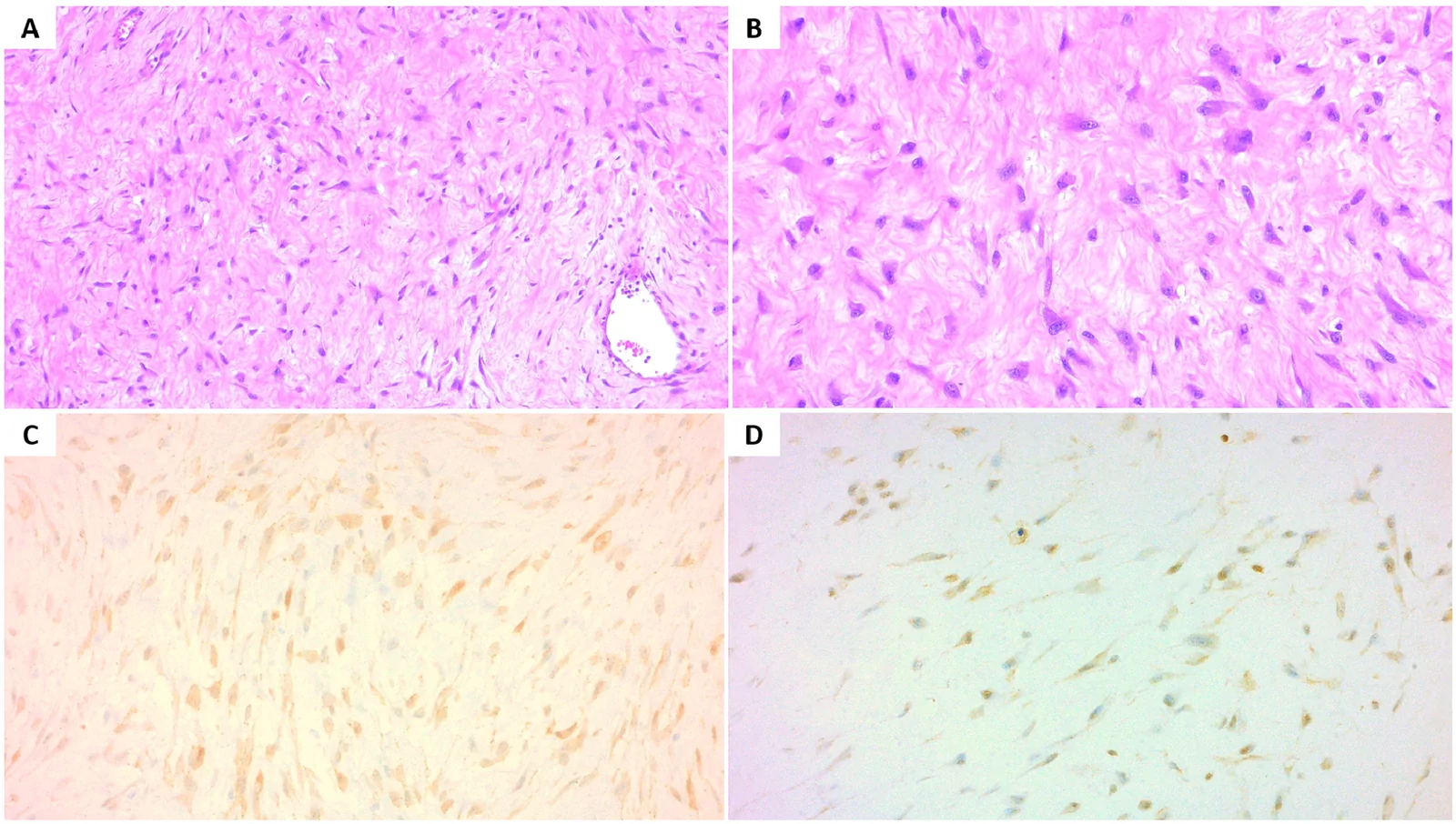
Histopathologic and immunohistochemical findings of desmoid-type fibromatosis. (A and B) Hematoxylin and eosin staining demonstrates fibroblastic and myofibroblastic spindle-cell proliferation with mild cytologic atypia, no significant mitotic activity, and abundant collagenous stroma. (C) β-catenin immunostaining shows diffuse nuclear positivity. (D) Smooth muscle actin immunostaining demonstrates cytoplasmic positivity.

Histopathologic examination revealed spindle-cell proliferation composed of fibroblasts and myofibroblasts with mild cytologic atypia and no significant mitotic activity. Immunohistochemical analysis demonstrated nuclear positivity for β-catenin and cytoplasmic positivity for smooth muscle actin, confirming the diagnosis of desmoid-type fibromatosis (Fig. 5).

**Fig. 6:**
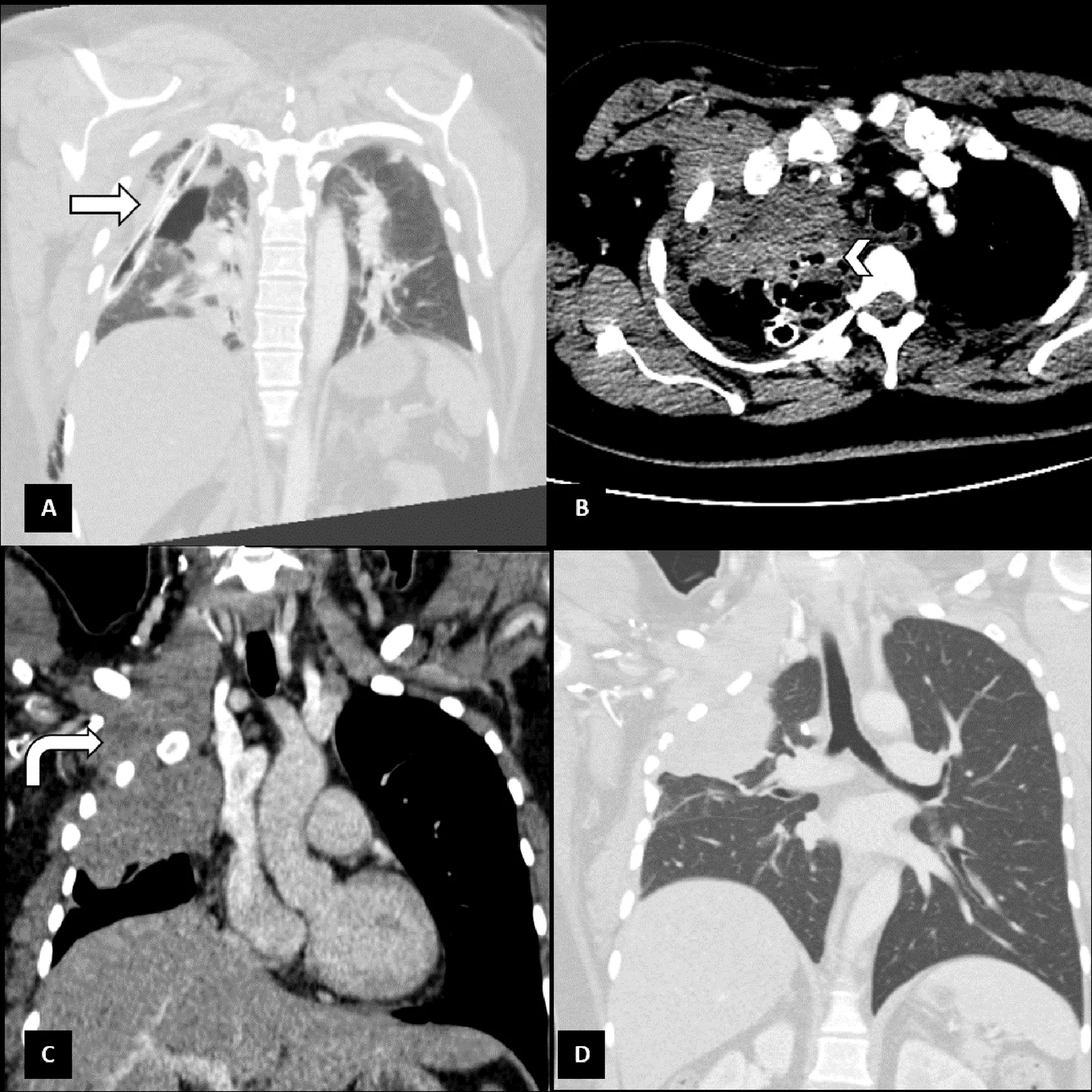
Postoperative and follow-up contrast-enhanced chest CT. (A and B) Images obtained 8 days after surgery. (A) Coronal lung window shows a chest tube and right pneumothorax (arrow). (B) Axial soft-tissue window demonstrates postoperative atelectasis (arrowhead) and residual tumor extending into adjacent soft tissues. (C and D) CT obtained 30 months later shows persistent and progressive residual disease. (C) Coronal image demonstrates involvement of the right superior thoracic inlet with sparing of the contralateral side. (D) Coronal lung window shows mass effect on the adjacent pulmonary parenchyma.

Following surgery, the patient experienced partial improvement of symptoms, particularly decreased pain and numbness. Early postoperative imaging demonstrated residual disease (Fig. 6A and B).

On long-term follow-up, 30 months after surgery, the patient returned with persistent numbness of the right upper extremity. Follow-up CT demonstrated a reduction in lung parenchymal compression due to a decrease in tumor size; however, there was persistent involvement of the adjacent soft tissues, particularly at the level of the superior thoracic inlet (Fig. 6C and D). Because the initial tumor removal was incomplete and performed in multiple fragments, negative surgical margins could not be achieved. The persistence of neurologic symptoms was considered likely secondary to tumor extension into the thoracic inlet with possible compression of adjacent neurovascular structures.

The case was reviewed by a multidisciplinary team to determine the most appropriate management strategy. Despite the emerging role of active surveillance and nonsurgical therapies in desmoid-type fibromatosis, surgical re-intervention was favored because of the persistent neurologic symptoms and the need for timely relief of the local mass effect. Radiotherapy was considered less suitable because of the unacceptable risk of toxicity to the adjacent intrathoracic and neurovascular structures. The patient also expressed a preference for definitive local treatment. At the time of the most recent evaluation, surgical planning was underway to decompress the affected neural structures and achieve maximal safe resection of the residual tumor.

## Discussion

Desmoid-type fibromatosis (DF) is strongly associated with dysregulation of the Wnt/β-catenin signaling pathway, which plays a fundamental role in cellular differentiation during embryogenesis and in tissue homeostasis and regeneration in adulthood [4]. β-catenin, regulated by the APC gene, is a key molecular component of this pathway and serves as a crucial diagnostic marker [4].

Macroscopically, desmoid tumors are typically firm, gray-white lesions resembling scar tissue. Histologically, they are characterized by a poorly defined proliferation of spindle cells with myofibroblastic differentiation embedded in an abundant collagenous stroma, usually without significant atypia, necrosis, or mitotic activity. Immunohistochemically, they demonstrate nuclear positivity for β-catenin along with expression of markers such as vimentin and smooth muscle actin, while lacking expression of S-100 and desmin [4]. These features are essential for distinguishing DF from other spindle-cell neoplasms.

A major diagnostic challenge arises in differentiating DF from other benign-appearing spindle-cell neoplasms, including inflammatory myofibroblastic tumors (IMTs), which can present with similar clinical and imaging features. IMTs most commonly occur in the lung but may arise in multiple anatomical sites [5]. Histologically, IMTs are characterized by spindle-cell proliferation within a myxoid-to-collagenous stroma accompanied by a prominent inflammatory infiltrate composed primarily of plasma cells and lymphocytes [5]. Unlike DF, immunohistochemistry in IMTs is less specific and not always required for diagnosis due to the variable expression and lack of specificity of myofibroblastic markers [5]. Imaging findings of IMTs are also heterogeneous and reflect their underlying histopathologic composition [6].

In the present case, the initial biopsy favored a benign spindle-cell tumor because of its bland cytologic appearance and absence of significant atypia, necrosis, or mitotic activity. However, these findings did not fully explain the infiltrative appearance, involvement of adjacent structures, and progressive behavior demonstrated on imaging. Histopathologic evaluation of the subsequently and incompletely excised tumor provided a more representative specimen, and nuclear positivity for β-catenin on immunohistochemistry established the diagnosis of desmoid-type fibromatosis. Thus, the correlation between the locally aggressive imaging features and the immunohistochemical findings was essential for resolving the initial diagnostic uncertainty.

As stated before, imaging evaluation plays a central role not only in diagnosis but also in treatment planning and follow-up [4]. Ultrasound, CT, and MRI are the main imaging modalities used. On ultrasound, lesions may range from well-defined masses to poorly defined infiltrative tumors, with characteristic signs such as the “tail sign” and “staghorn sign,” reflecting extension along fascial planes [4].

On CT, desmoid tumors may appear as well-defined or infiltrative soft-tissue masses with attenuation similar to skeletal muscle, showing variable enhancement depending on their fibrotic or myxoid composition [4]. These imaging features are often nonspecific and may mimic malignant lesions, as demonstrated in the present case.

MRI is the imaging modality of choice for extra-abdominal desmoid tumors and provides superior soft-tissue characterization. Signal intensity varies depending on the relative proportion of collagen and cellular components. Lesions with higher collagen content typically demonstrate low signal intensity on both T1- and T2-weighted images, whereas more cellular or myxoid tumors appear more heterogeneous with higher T2 signal. Restricted diffusion may also be observed and is thought to correlate with increased cellularity, although this finding remains nonspecific [6]. Characteristic findings include the “band sign,” representing nonenhancing low-signal linear bands [7,8], and the “fascial tail” sign, reflecting tumor extension along fascial planes [9].

Neurologic symptoms such as numbness or paresthesia may occur due to involvement of adjacent neurovascular structures, particularly in lesions with chest wall and thoracic inlet extension, as illustrated in this case.

Management of desmoid tumors remains challenging due to their locally aggressive behavior. Surgical resection is considered a potentially curative option; however, complete excision with negative margins is often difficult, especially in anatomically complex regions [3]. Despite treatment, local recurrence rates range from 20% to 65% within 5 years, particularly in cases with large tumor size, extra-abdominal location, or positive margins [3].

Alternative treatment strategies include active surveillance, radiotherapy, systemic therapy, and targeted agents, with management decisions tailored to tumor location, progression, and clinical symptoms [1]. In some cases, tumor stabilization or regression may occur without intervention, supporting a more conservative approach in selected patients [8].

In the present case, incomplete resection and involvement of critical structures likely contributed to tumor persistence and progression. Follow-up imaging demonstrated reduction in pulmonary compression but persistent soft-tissue involvement at the thoracic inlet, correlating with ongoing neurologic symptoms and highlighting the importance of long-term surveillance and multidisciplinary management.

## Conclusion

Pleural desmoid tumors are rare, locally aggressive lesions that may mimic inflammatory myofibroblastic tumors both clinically and radiologically, posing a significant diagnostic challenge. Accurate diagnosis relies on histopathologic and immunohistochemical correlation, particularly the demonstration of β-catenin positivity. Recognition of their infiltrative behavior, potential involvement of critical structures such as the thoracic inlet, and high risk of recurrence is essential to guide appropriate management and long-term follow-up.

## Declaration of generative AI and AI-assisted technologies in the working process

During the preparation of this work, the authors used ChatGPT (OpenAI) to assist with translation of the manuscript and to review grammar and spelling. After using this tool, the authors carefully reviewed and edited the content as needed and take full responsibility for the content of the published article.

## Patient consent

Written informed consent was obtained from the patient for publication of this case report and accompanying images. The authors confirm that a copy of the written consent is available for review by the journal upon request.
